# Expression of Collagen (Types I, III, and V), HSP47, MMP-2, and TIMP-1 in Retrobulbar Adipose Tissue of Patients with Thyroid-Associated Orbitopathy

**DOI:** 10.1155/2020/4929634

**Published:** 2020-04-21

**Authors:** Hongyang Luo, Taixiang Liu, Huasheng Yang, Huijing Ye, Xin Luo

**Affiliations:** ^1^Guizhou Ophthalmic Hospital, The Affiliated Hospital of Zunyi Medical College, Zunyi 563003, China; ^2^Zhongshan Ophthalmic Center, Sun Yat-Sen University, Guangzhou 510003, Guangdong Province, China

## Abstract

**Objective:**

This study aimed to investigate the expression of collagen (types I, III, and V), heat shock protein 47 (HSP47), matrix metalloproteinase-2 (MMP-2), and tissue inhibitors of metalloproteinase-1 (TIMP-1) in the retrobulbar adipose tissues of patients with thyroid-associated orbitopathy (TAO).

**Materials and Methods:**

The retrobulbar adipose tissues were collected from 4 TAO patients undergoing orbital decompression and 4 ocular enucleation patients with atrophic eyeball caused by ocular trauma between May 2019 and September 2019. Masson staining was performed to analyze the differences in collagen expression and degree of histologic fibrosis in each sample. The protein expressions of collagen (types I, III, and V), HSP47, MMP-2, and TIMP-1 were determined by western blotting. The data of western blotting were analyzed using SPSS version 17.0, with independent *t*-tests.

**Results:**

The results of Masson staining showed that the expression of collagen fibers in the TAO group was significantly higher than that in the control group, and the fibers were diffuse and irregular in distribution. The expression level of collagen (types I, III, and V), HSP47, MMP-2, and TIMP-1 in the TAO group were significantly higher than that in the control group (*P* < 0.05).

**Conclusion:**

The proliferation and fibrosis of retrobulbar adipose tissue in TAO patients might be related to the increased expression of collagen (types I, III, and V) and HSP47 and decreased degradation of extracellular matrix.

## 1. Introduction

Thyroid-associated orbitopathy (TAO) is one of the most common diseases of the orbit, with an incidence of about 20% in adults. This disease not only affects the appearance of patients but also causes visual impairment or even blindness due to exophthalmos, diplopia, exposure keratopathy, and compressive optic neuropathy. Many patients have severe pain associated with TAO, seriously affecting the work and life of the patients [1].

TAO involves the pathological process of fibrosis, and it is associated with abnormal accumulation of extracellular matrix. This is attributed by the accumulation of extracellular matrix, especially collagen, leading to tissue proliferation, hardening, or scarring [2]. However, the mechanism of abnormal accumulation of extracellular matrix in orbital fat tissues in TAO patients is still unknown. Abnormal metabolism of collagen might be associated with it, as collagen is regarded as a major component of extracellular matrix.

Heat shock protein 47 (HSP47) is a procollagen/collagen-specific molecular chaperone protein that is associated with abnormal collagen synthesis, and it can be expressed in nearly all types of cell-expressed collagen proteins [3]. HSP47 has unique substrate specificity in identifying the Pro-Arg-Gly sequence in the Gly-x-y sequence of collagen in the endoplasmic reticulum, especially Arg (arginine), and then, it binds to the newly synthesized procollagen to maintain the stable structure of collagen triple helix [4]. Several diseases are directly related to the abnormal expression of HSP47, clinically, and abnormal accumulation of collagen induced by HSP47 overexpression acts as a risk factor for fibrosis of tissues and organs. Naitoh et al. [5] reported that the mRNA expression of type I and type III collagen in scar tissues were 20 times higher than that in normal tissues, and subsequently, the mRNA and protein levels of HSP47 were upregulated 8 and 16 times, respectively. A study reported that the expression of collagen types I and III and HSP47 were increased, and the matrix metalloproteinases (MMPs) and tissue inhibitors of metalloproteinases (TIMPs) led to abnormal expression during the process of conjunctival matrix remodeling in a patient with epidermolysis bullosa acquisita (EBA) [6]. Previous clinical and experimental research studies indicated that high expression of HSP47 and abnormal expression of enzymes that maintain extracellular matrix balance (MMPs and TIMPs) probably showed an association with collagen proliferation and fibrotic process. Currently, the role of their expression in retrobulbar adipose tissues of TAO patients remains unexplored. Therefore, this study aimed to evaluate the expression of collagen (types I, III, and V), HSP47, MMP-2, and TIMP-1 in retrobulbar adipose tissues of patients with TAO and whether they play a role in tissue fibrosis and process of hyperplasia.

## 2. Materials and Methods

### 2.1. Participants and Specimen Collection

#### 2.1.1. Patient Characteristics

From May 2019 to September 2019, 4 TAO patients (TAO group) who underwent orbital decompression to relieve their ocular symptoms which are mainly caused by the proliferation and fibrosis of retrobulbar adipose tissue, at the Ophthalmic Center of Zhongshan Hospital, were labeled as A, B, C, and D, respectively. Four patients (control group) who underwent ocular enucleation of atrophic eyeball caused by ocular trauma, in which TAO and Graves' disease (GD) were explicitly excluded, were labeled as a, b, c, and d, respectively.

#### 2.1.2. Collection of Retrobulbar Adipose Tissue

The retrobulbar adipose tissue samples were collected by surgical resection of the patients and stored at −80°C in a refrigerator after quick-freezing using liquid nitrogen. The frozen tissues were used for western blotting. The remaining tissues were fixed in 4% paraformaldehyde for Masson staining.

The present study was approved by the Ethics Committee of Zunyi medical university. Each patient provided signed written informed consent.

### 2.2. Masson Staining

The retrobulbar adipose tissues were dehydrated using graded ethanol, and then, they were embedded in paraffin. After paraffin solidification, the specimens were sectioned at 4 *μ*m thickness and dried in a drying oven. The paraffin sections were dewaxed and stained with potassium dichromate, ferro sulfonate, lichen red acid magenta, phosphomolybdate, and aniline blue. The stained sections were differentiated using glacial acetic acid and anhydrous ethanol. Finally, the slices were treated with anhydrous ethanol and xylene, and then, they were sealed with neutral gum. The samples were photographed and analyzed by light microscopy (Nikon Eclipse E100, Nikon, Japan).

### 2.3. Western Blot

Western blotting was performed to detect the expression of collagen (types I, III, and V), HSP47, MMP-2, and TIMP-1.

The total proteins of the tissue samples were extracted using RIPA lysis buffer after washing and mechanical cutting. The protein content of the tissue samples, after measuring, underwent sodium dodecyl sulfate-polyacrylamide gel electrophoresis (SDS-PAGE) for 4-5 h at a voltage of 40 V and was stopped when bromophenol blue ran out. The proteins were then transferred into an electro-transfer tank at a voltage of 60 V for 2 h. The blots were incubated overnight at 4°C with primary antibodies (1 : 1000), followed by washing the membranes and blotting with secondary antibody (1 : 1000) for 1.5 hours at room temperature. Finally, the gray-scale value of proteins was measured by Image J software after the protein bands were detected by chemiluminescence (Tanon 1220, Tanon, China). The antibodies against collagen III and MMP-2 were purchased from Abcam (Cambridge, MA, USA). The antibodies against collagen I were obtained from the Boster Biological Technology. The antibodies against collagen V were obtained from LifeSpan BioSciences (Seattle, USA). The antibodies against HSP47 were obtained from Novus Biologicals (Littleton, Colorado, USA), and the antibodies against TIMP-1 were obtained from Proteintech Group (Chicago, Illinois, USA). GAPDH was used as the negative control.

### 2.4. Statistical Analysis

SPSS software, version 17.0, was used for performing statistical analyses. The normality of the data was first verified by the Kolmogorov–Smirnov test of normality. All results were normally distributed and an independent *t*-test was used for comparing the experimental group and the control group. A significance level of 0.05 was set for all statistical tests.

## 3. Results

### 3.1. Masson Staining

**Figure 1:**
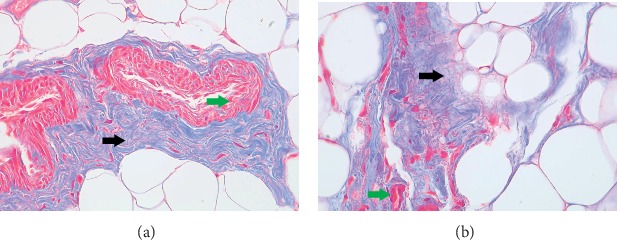
The expression of collagen in the retrobulbar adipose tissue as measured by Masson's staining. In Masson's staining, the collagen was stained blue (black arrow) and cardiomyocytes were stained red (green arrow). (a) Control group. (b) TAO group. The magnification is ×400.

The tissue slices that underwent Masson staining (Figure 1) showed that the retrobulbar adipose tissues were completely with clear cell shape and the collagen fibers showed regular organization in the control group. However, the tissue structure of the TAO group remained vague, and the collagenous fibers were disorganized and showed diffused distribution. The expression of collagen fibers was significantly higher than that of the control group.

### 3.2. Protein Expression of Collagen (Types I, III, and V), HSP47, MMP-2, and TIMP-1 in Retrobulbar Adipose Tissues of Patients with TAO

**Figure 2:**
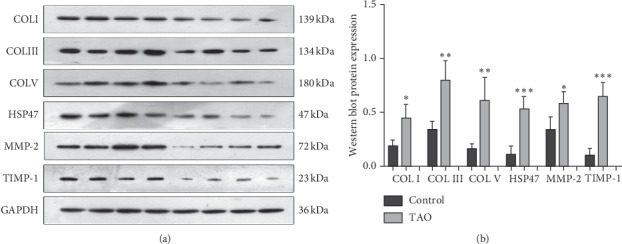
The protein expression of collagen (types I, III, and V), HSP47, MMP-2, and TIMP-1 were increased in patients with TAO. (a) Western blotting was performed to detect the protein expression of collagen (types I, III, and V), HSP47, MMP-2, and TIMP-1. (b) Gray value analysis of the two groups. ^*∗*^*P* < 0.05 vs. NC; *n* = 4. ^*∗∗*^*P* < 0.01 vs. NC; *n* = 4. ^*∗∗*^*P* < 0.001 vs. NC; *n* = 4.

**Figure 3:**
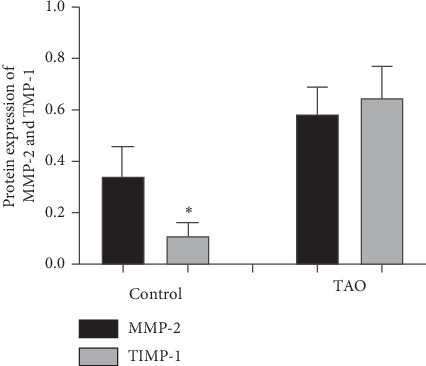
Gray value analysis of western blotting in the control group and TAO group.^*∗*^*P* < 0.05 vs. NC; *n* = 4.

The results of western blotting demonstrated that the protein expression of collagen (types I, III, and V), HSP47, MMP-2, and TIMP-1 (Figure 2) was significantly increased in the TAO group when compared to the control group (*P* < 0.05). The results showed no significant differences in the expression of collagen types in either the control group or the TAO group, while the expression characteristics of MMP-2 and TIMP-1 in the two groups showed differences. Further analysis revealed that the expression level of MMP-2 was significantly higher than TIMP-1 in the control group (Figure 3), while the expression level of TIMP-1 in the TAO group (about 5 times that of the control group) was significantly higher than the increase in MMP-2 (about 0.7 times that of the control group).

## 4. Discussion

TAO is a common autoimmune disease, and approximately 85% of patients develop ocular signs and symptoms within 18 months [7]. Fibrosis is regarded as an important pathological process of TAO, and hyperplasia of orbital connective tissue and increased volume of extraocular muscle might be the main cause for ocular clinical symptoms [8, 9]. The pathological process which mainly includes proliferation of fibroblasts, increased deposition of extracellular matrix, and adipocyte differentiation and proliferation leading to edema, enlargement of extraocular muscles, and increased volume of orbital soft tissues results in exophthalmos [10].

The fibrotic process is usually closely related to the abnormal accumulation of extracellular matrix. Reduction in the deposition of extracellular matrix alleviates ocular symptoms including eye irritation in patients with TAO, but the mechanism of abnormal deposition of extracellular matrix still remains unclear. Collagen is one of the main components of extracellular matrix, and abnormal metabolism of it might lead to the deposition of extracellular matrix. For example, deposition of a large amount of collagen and extracellular matrix was found in fibrotic lung tissues [11]. Our study results demonstrated that the expression level of collagen (types I, III, and V) was significantly increased in the TAO group when compared with the control group, showing no significant difference in the expression among the three types of collagen. Therefore, the fibrosis of retrobulbar adipose tissue in TAO patients was speculated to be related to the abnormal accumulation of collagen, and it leads to the proliferation of tissues eventually.

Our results also showed that the protein levels of HSP47 in retrobulbar adipose tissues of TAO patients were significantly higher than that in the control group. It has been reported that the collagen synthesis is closely associated with HSP47 [3]. All the cells can express collagen and HSP47, and in addition, HSP47 concentration is also related to collagen. Many studies have confirmed that HSP47 is involved in collagen biosynthesis, structural assembly, maturation, and its secretion. First, HSP47 prevents the formation of invalid polymer during the early stage of procollagen chain, modifying it further. Second, HSP47 prevents degradation and triple helix structure polymerization of procollagen, promoting the secretion of procollagen [12–14]. The abnormalities of HSP47 metabolism might cause abnormal synthesis and secretion of collagen, which is considered as a key factor in regulating the collagen contents in tissues. Therefore, we speculated that the increased expression of HSP47 in retrobulbar adipose tissues of TAO patients might be related to the increased collagen contents and fibrosis in the tissues.

Furthermore, the high expression of HSP47 showed possible association with inflammatory factors produced by autoimmune inflammation in TAO patients. The expression of TGF-*β* in retrobulbar adipose tissues of TAO patients was significantly higher than that in normal tissues [11]. In conjunctiva, lung, and liver tissues, cytokines such as TGF-*β*, IL-4, and IL-1 promoted collagen synthesis as well as its deposition by regulating the expression of HSP47 to promote fibrosis of tissues and organs [15–18]. In addition, cell experiments reported that HSP47 expression showed positive association with TGF-*β* [19], but whether similar mechanism exists in the fibrosis of retrobulbar adipose tissue in TAO patients has not yet been reported. Since our specimens were collected during surgery, patients with TAO underwent routine treatment with methylprednisolone (at a dose of 500–1000 mg, once a day for 3 days) to reduce the intraoperative risk and postoperative inflammatory response, which in turn might lead to the changes in the expression of inflammatory factors. Previous studies have revealed that the expression of collagen, IL-1, IL-2, IL-4, TGF-*β*, and other inflammatory factors is decreased after using high-doses of methylprednisone for femoral head necrosis, shoulder joint injury, spinal cord injury, Bell's palsy, and other diseases [20–24]. By considering the patient's health and ethical issues, we did not design the TAO surgery group without anti-inflammatory treatment to verify the relationship between HSP47 and inflammatory factors in the retrobulbar adipose tissues. Consequently, we explored whether the overexpression of HSP47 in retrobulbar adipose tissues of TAO patients was related to increased inflammatory factors in subsequent cell experiments and further analyzed the possible pathological mechanism of fibrosis.

The balance of extracellular matrix is maintained by two enzymes: MMPs and TIMPs. MMPs, also known as stroma, degrade the extracellular matrix, while TIMPs inhibit the expression of MMPs, which maintain the homeostasis of extracellular matrix. The expression level of TIMP-1 was increased in fibrous tissues, and the MMP-2/TIMP-1 ratio was decreased gradually during the formation of scar tissue in mice [2, 25, 26]. Our experimental results revealed that the expressions of MMP-2 and TIMP-1 were higher in the TAO group than those in the control group. Moreover, the increased levels of TIMP-1 were higher than that of MMP-2. From these data, we deduced that TIMP-1 inhibited degradation of the extracellular matrix by MMP-2, leading to the deposition of the extracellular matrix in patients with TAO.

Currently, glucocorticoids (GC) remain the top choice for treating TAO. However, GC often cause gastrointestinal injury, osteoporosis, and other adverse reactions while undergoing treatment but relieve the symptoms of eye discomfort. Therefore, it is imperative to search for an ideal drug that can effectively treat TAO with fewer side effects. The results of this study revealed that the increased expression of HSP47 in retrobulbar adipose tissues of TAO patients might be related to tissue fibrosis. Furthermore, exploration on this topic might provide a new target and theoretical basis for the prevention and treatment of retrobulbar adipose tissue fibrosis in TAO patients.

## Data Availability

All data generated or used during the study are available from the corresponding author upon request.
